# Insight into a molecular interaction force supporting peptide backbones and its implication to protein loops and folding

**DOI:** 10.1080/07391102.2014.984333

**Published:** 2014-12-22

**Authors:** Qi-Shi Du, Dong Chen, Neng-Zhong Xie, Ri-Bo Huang, Kuo-Chen Chou

**Affiliations:** ^a^ State Key Laboratory of Non-food Biomass and Enzyme Technology, National Engineering Research Center for Non-food Biorefinery, Guangxi Academy of Sciences, 98 Daling Road, Nanning, Guangxi530007, China; ^b^ Nanning Fermentation and Enzyme Engineering Research Center, Life Science and Biotechnology College, Guangxi University, Nanning, Guangxi530004, China; ^c^ Center of Excellence in Genomic Medicine Research (CEGMR), King Abdulaziz University, Jeddah21589, Saudi Arabia; ^d^ Gordon Life Science Institute, 53 South Cottage Road, Belmont, MA02478, USA

**Keywords:** protein structures, secondary structure, protein loops, Hp–*π* interaction, hydrogen bond

## Abstract

Although not being classified as the most fundamental protein structural elements like *α*-helices and *β*-strands, the loop segment may play considerable roles for protein stability, flexibility, and dynamic activity. Meanwhile, the protein loop is also quite elusive; i.e. its interactions with the other parts of protein as well as its own shape-maintaining forces have still remained as a puzzle or at least not quite clear yet. Here, we report a molecular force, the so-called polar hydrogen–*π* interaction (Hp–*π*), which may play an important role in supporting the backbones of protein loops. By conducting the potential energy surface scanning calculations on the quasi *π*-plane of peptide bond unit, we have observed the following intriguing phenomena: (1) when the polar hydrogen atom of a peptide unit is perpendicularly pointing to the *π*-plane of other peptide bond units, a remarkable Hp–*π* interaction occurs; (2) the interaction is distance and orientation dependent, acting in a broad space, and belonging to the ‘point-to-plane’ one. The molecular force reported here may provide useful interaction concepts and insights into better understanding the loop’s unique stability and flexibility feature, as well as the driving force of the protein global folding.

## 1. Introduction

**Figure 1.**
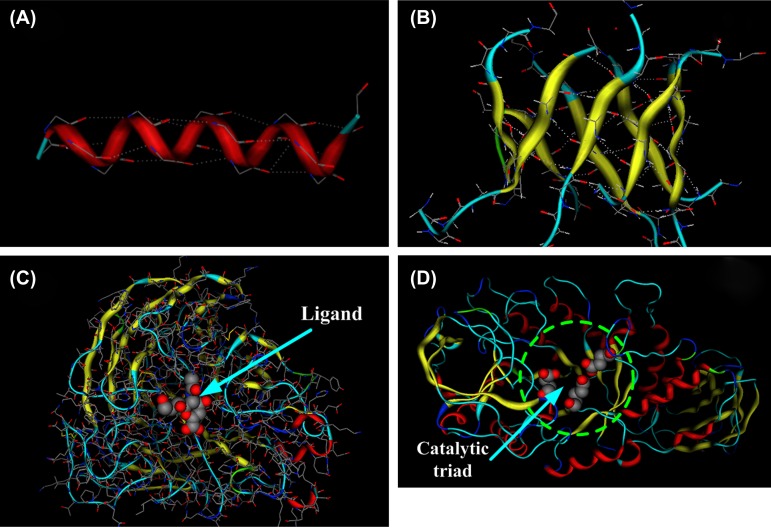
The structures of protein secondary structures *α*-helices, *β*-strands, and loops. (A) The hydrogen bonds in *α*-helix. In *α*-helix, the *π*-planes of peptide bond units in the up cycle and the lower cycle are arranged in parallel, and the hydrogen bond donors and acceptors between two cycles are in the best orientation for hydrogen bonds. (B) The hydrogen bonds in TIM (*β*/*α*)_8_ barrel of *α*-amylase. In *β*-strand, all *π*-planes of peptide bond units are arranged in parallel. The hydrogen bond donors and acceptors between two neighboring *β*-strands form stable hydrogen bonds. (C) The ligand binding location in neuraminidase (PDB code 1F8B) of influenza A viruses. All binding sites are in the loops. (D) In *α*-amylase (pdb code: 1BLI), three key residues (Asp231, Glu261, and Asp328), the so-called the catalytic triad, are located at the loops. In average, the residues in loops are up to 47%.

More than 60 years ago, the segmental structures of *α*-helices and *β*-strands in proteins were first reported and described, rather than by X-ray or NMR experimental observations, but by Pauling and Corey (1951; Pauling, Corey, & Branson, 1951) using the logical deduction based on the concept of hydrogen bond interactions. In justifying their hypothetical structures, Pauling and Corey considered which conformations of peptides were sterically allowed and which kind of hydrogen bond distribution therein is most efficient. Under the light of hydrogen bond concept, Pauling and Corey designed the structures of *α*-helices and *β*-strands, in which the hydrogen bonds maintain the structures of these two kinds of regular segments effectively, as shown in Figure 1(A) and (B). In the *α*-helices, the up cycle and the low cycle of peptide chains are fixed by the hydrogen bonds, and in the *β*-strands, the two neighboring *β*-sheets are also fixed by the hydrogen bonds. About three decades later, the handedness of *α*-helix, *β*-sheet/barrel formed by *β*-strands, as well as their packing arrangements in proteins have been in-depth investigated from the energetic point of view by counting various molecular forces such as hydrogen bond interaction, electronic interaction, and van der Waals interaction, as elaborated in a series of publications during the 80’s of last century (see e.g. Chou & Scheraga, 1982; Chou, Nemethy, & Scheraga, 1983a, 1983b, 1984; Chou, Nemethy, Pottle, & Scheraga, 1985; Chou, Nemethy, Rumsey, Tuttle, & Scheraga, 1985, 1986; Chou, Pottle, Nemethy, Ueda, & Scheraga, 1982).

According to the protein structural architecture, the loops are not classified into the regular category. But so far the efforts of using the energetics or molecular forces to analyze the protein secondary structure are successful only for the regular structure elements (Carlacci & Chou, 1990b, 1991; Chou, 1992; Chou, Carlacci, & Maggiora, 1990; Chou, Carlacci, Maggiora, Parodi, & Schulz, 1992; Chou, Maggiora, Nemethy, & Scheraga, 1988; Chou, Nemethy, Pottle, & Scheraga, 1989; Chou, Nemethy, & Scheraga, 1990; Chou et al., 1990; Gerritsen, Chou, Nemethy, & Scheraga, 1985; Scheraga, Chou, & Nemethy, 1982). Actually, in proteins about 47% of all residues belong to this type of irregular structural segments (the loops or coils) (Gordon, Marshall, & Mayot, 1999; Harrison, Pearl, Mott, Thornton, & Orengo, 2002; Joseph, Srinivasan, & de Brevern, 2011; Krissinel & Henrick, 2004; Lu, 2000). Particularly, when using energetic approach to model many drug-target proteins important for drug development such as bovine somatotropin (Carlacci, Chou, & Maggiora, 1991), one could not avoid dealing with loop structures (Carlacci et al., 1991). Unfortunately, so far we still do not know the interaction force in supporting the loop structures.

In many cases, the loops are the most active parts in enzymes that play a key role in biological functions (Sirois, Sing, & Chou, 2005; Sirois, Touaibia, Chou, & Roy, 2007; Wen, Wang, Yang, & Xie, 2010, 2011). For example, almost all drug binding sites in neuraminidase (NA) of influenza A viruses (PDB code 1F8B) (Palese, Tobita, Ueda, & Compans, 1974; von Itzstein, 2007) are in the loops, as shown in Figure 1(C). In Figure 1(D) the three key residues (Asp231, Glu261, and Asp328) of enzyme *α*-amylase (PDB code 1BLI) (Machius, 1998), the so-called the catalytic triad, are located in the loops. The structural changes in the 150 loop of NA may cause the drug resistance problem (Du, Wang, & Huang, 2010; Du, Wei, & Huang, 2011; Wang, Du, Huang, & Zhang, 2009). The loops also play a key role for the two most remarkable features in proteins; i.e. the stability and flexibility, such as in stabilizing the helix bundles (Carlacci & Chou, 1990a; Chou, 1991; Chou & Zheng, 1992; Chou, Maggiora, & Scheraga, 1992; Thompson, Chou, & Zheng, 1995), beta-barrel (Chou & Carlacci, 1991), and ion-channels (Berardi, Shih, Harrison, & Chou, 2011; OuYang et al., 2013; Schnell & Chou, 2008), and allowing the occurrence of low-frequency internal collective motions responsible for many remarkable biological functions of proteins (Chou, Li, Klee, & Bax, 2001; Chou, 1983, 1985, 1987, 1988, 1989; Chou & Chen, 1977; Wang & Chou, 2009, 2010; Wang, Gong, Wei, & Li, 2009). Proteins (or enzymes) are not a rigid object, but an entity that is dynamically quite flexible and structurally relatively stable in order to be able to perform various remarkable biological functions. In contrast, the *α*-helices, *β*-strands, and tight turns are relatively rigid parts, although they may move with respect to one another via modulating the conformation of the relevant loop. In other words, the loop can be likened to the ‘pivot’ or ‘hinge,’ around which the rigid parts of a protein may move to realize its function without breaking the system owing to the loop’s existence.

The knowledge of the interaction forces in protein irregular structures is the necessary theoretical basis for development of computational methods and tools to predict the protein folding and 3D structures. In the study of protein family evolution, most structural changes happen in the loops (Agarwal, Rajavel, Gopal, & Srinivasan, 2009; Bhaduri, Pugalenthi, & Sowdhamini, 2004; Marti-Renom, Ilyin, & Sali, 2001; Mizuguchi, Deane, Blundell, & Overington, 1998; Sujatha, Balaji, & Srinivasan, 2001). The loops are often the main engineering targets in protein engineering and protein design for improving the bioactivities and physicochemical properties of enzymes (Kuhlman et al., 2003).

Unlike *α*-helices and *β*-strands, in the protein loops, the quasi *π*-planes of peptide bond units often form the dihedral angles around 90°, which are not the appropriate orientations for the hydrogen bonds. The structures of loops cannot get reasonable explanation using the concept of hydrogen bonds. In the three-dimensional structures of proteins the loops look like soft silk threads. Are there any supporting forces in the loops? What physicochemical factors maintain the structural shapes of loops? In this study, we are to explore this important question based on the concept of polar hydrogen–*π* interactions (Birchall et al., 2011; Li & Fan, 2010; Mróz, 1995; Pidaparti, Svintradze, Shan, & Yokota, 2009), evaluate the interaction energies and analyze the role of polar hydrogen–*π* interactions in loops using quantum chemical calculations at the higher level.

## 2. Method and theory

Hydrogen–*π* bonds are a special molecular interaction type, referring to the interactions between hydrogen atoms (attaching to different atomic groups) and the *π*-electron density of conjugate molecules (or groups). Hydrogen–*π* interactions can be classified into two types: the non-polar hydrogen–*π* interactions, in which the hydrogen atoms are attached to the carbon atoms (H–*π* or CH–*π*) (Birchall et al., 2011; Pidaparti et al., 2009); and the polar hydrogen–*π* interactions, in which the hydrogen atoms are attached to the electronegative atoms, such as oxygen, nitrogen, and chlorine (Birchall et al., 2011; Hughes & Waters, 2006). The polar hydrogen–*π* bonds and non-polar hydrogen–*π* bonds have different physical nature and properties. In this study, the notation ‘Hp-*π*’ is used for the polar hydrogen–*π* interactions (or bonds), to make it different from the common hydrogen bond (H–b) as well as the disputed non-polar hydrogen–*π* (H–*π* or CH–*π*) interactions. In protein structures, the Hp–*π* interactions could happen between amino acid side chains, or play roles in the peptide backbone of proteins. The Hp–*π* interactions between amino acid side chains have been studied in reference (Du, Wang, Du, Chen, & Huang, 2013). This study focuses on the Hp–*π* interactions in peptide backbones of proteins.

**Figure 2.**
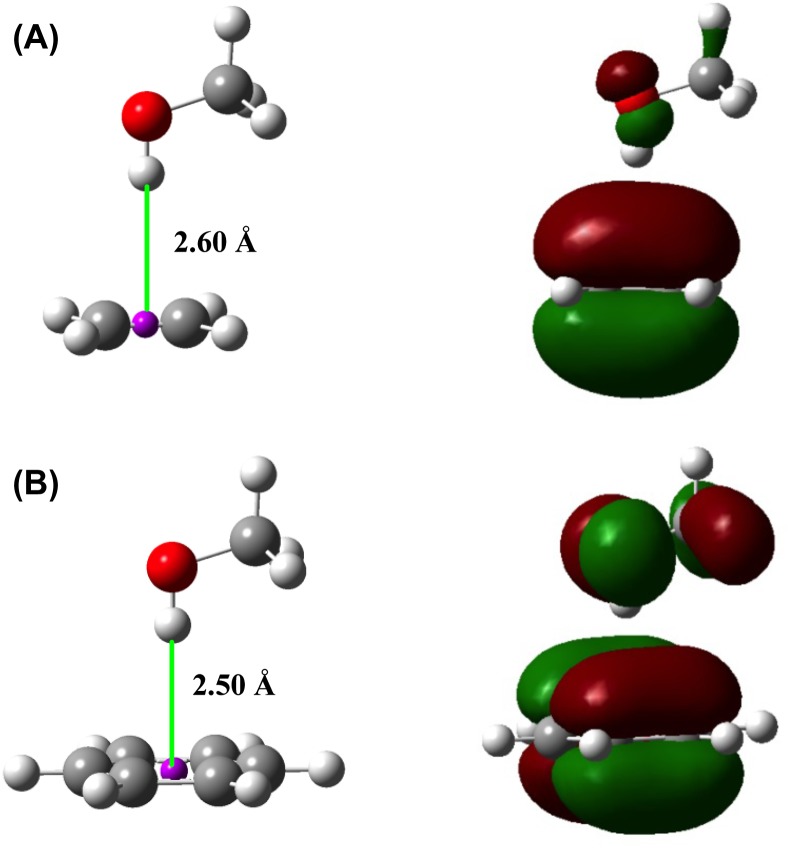
Typical polar hydrogen–*π* (Hp–π) interactions. (A) The Hp–π interaction between CH_3_OH and C_2_H_4_ (−12.887 kJ/mol). The polar hydrogen atom of CH_3_OH perpendicularly points to the center (pink ball) of double bond of C_2_H_4_. (B) The Hp–π interaction between CH_3_OH and C_6_H_6_ (−22.731 kJ/mol). The polar hydrogen atom of CH_3_OH perpendicularly points to the center of benzene *π*-plane (pink ball). The polar hydrogen atom is in close touching with the *π*–MO of aromatic molecules.

The peptide bond units of protein backbones are *π*-groups comprised by atom N, C, and O. The peptide bond units possess the interaction elements for both common hydrogen bond interactions and the polar hydrogen–*π* interactions. Although the hydrogen–*π* interactions have been studied for many years by many investigators (Birchall et al., 2011; Hughes & Waters, 2006; Pidaparti et al., 2009), the roles of Hp–*π* interactions in protein backbone structures and in protein folding have not been studied sufficiently. Two simple examples of the Hp–*π* interaction structures and highest occupied molecular orbital (HOMOs) of CH_3_OH…C_2_H_4_ and CH_3_OH…C_6_H_6_ are shown in Figure 2(A) and (B), where the polar hydrogen atom of CH_3_OH is perpendicularly pointing to the *π*-plane of the conjugate molecules. In Figure 2, the HOMOs of the two Hp–*π* interaction systems (CH_3_OH…C_2_H_4_ and CH_3_OH…C_6_H_6_) are shown

The electron dispersion is an important contribution in the Hp–*π* interaction energies (Birchall et al., 2011). For better evaluating the electron dispersion and correlation energies involved in the Hp–*π* interactions, the post Hartree-Fock methods (Cramer & Bickelhaupt, 2003; Sherrill & Schaefer III, 1999) are necessary. In this study, the QM methods coupled-cluster singles and doubles (CCSD) and coupled-cluster singles, doubles, and partial triples (CCSD(T)) (Lee & Rice, 1988; Purvis III & Bartlett, 1982; Sherrill & Schaefer III, 1999) are adopted. It essentially takes the basic Hartree–Fock molecular orbital method and constructs multi-electron wave functions using the exponential cluster operator to account for electron correlation and dispersion. This method has been used by some of the most accurate calculations for small- to medium-sized molecules (Agarwal et al., 2009; Cramer & Bickelhaupt, 2003; Scuseria & Schaefer III, 1989; Scuseria, Janssen, & Schaefer, 1988; Sindzingre, Lhuillier, & Fouet, 2001). On the other hand, since the Hp–*π* bond length (~2.5 Å) is much longer than the common hydrogen bonds (~2.0 Å), large basis sets are necessary, including the polarization functions, diffuse functions, and floating functions. The CCSD(T) is better than the CCSD, because the CCSD uses only single- and double-excited configurations, while the CCSD(T) also includes part triple-excited configurations. The large basis functions make the number of higher excited configurations increasing tremendously. Accordingly, the CPU-time of CCSD(T) calculations is much longer than that of the CCSD calculations. The state-of-the art method CCSD(T) with large basis set is extremely expensive and CPU-time consuming. The test calculation results of a small Hp–*π* interaction pair CH_3_OH…C_2_H_4_ by means of various methods and basis sets are listed in Table 1.

**Table 1.** Comparison of three methods (DFT, CCSD, and CCSD(T)) and four basis sets (6-311 + G(d,p), TZVP, cc-pVTZ, and 6-311 + G(d,p)+H-Bq) in calculations of polar hydrogen–*π* (Hp–*π*) interactions.

Hp–*π*	B3LYP		CCSD		CCSD(T)	
^a^CH_3_OH…C_2_H_4_	Energy (kJ/mol)	cpu-Time (h)	Energy (kJ/mol)	cpu-Time (h)	Energy (kJ/mol)	cpu-Time (h)
6-311G(d,p)	−7.29	0.1	−9.72	4.3	−10.748	14
TZVP	−7.658	0.05	−9.179	3	−10.105	7.6
cc-pVTZ	−8.269	0.2	−10.662	16.5	−11.853	44.6
^b^6-311Gdp+H-Bq	−7.547	0.1	**−11.715**	5.3	**−12.887**	17.9

^a^ Structures of molecular monomers are optimized at CCSD/6-311 + G(d,p) level.

^b^ A hydrogen ‘ghost atom’ (H-Bq) is added to the 6-311 + G(d,p) basis functions of polar hydrogen atom. The ghost atom H-Bq is in the middle of Hp–*π* bond, and the distance to polar hydrogen is 0.8 Å.

The Hp–*π* interaction energies, calculated using density functional theory (DFT) method B3LYP, are around 20–30% smaller than that of the two post Hartree-Fock methods (CCSD and CCSD(T)). This is because the common DFT methods fail in evaluating the dispersion energies that is an important contribution in Hp–*π* interactions. The more advanced method CCSD(T) gives better results than that of the CCSD method. However, the cpu-time of CCSD(T) is much longer than that of the CCSD. In solving this problem, a simple method is to use ‘ghost atoms.’ The ghost hydrogen atom H-Bq is an empty atom possessing the basis functions of hydrogen atom, but having no nucleus charge and electron (Asturiol, Duran, & Salvador, 2008; Balabin, 2008; Mayer & Valiron, 1998). As shown in Table 1, the CCSD calculation using 6-311 + G(d,p) basis set plus a hydrogen ‘ghost atom’ H-Bq yields the result (−11.715 kJ/mol), very close to the result (−11.853 kJ/mol) of the CCSD(T)/cc-pVTZ method. However, the cpu-time taken by CCSD calculation is only 1/8 of the time by CCSD(T) calculation that is from 5.3 to 44.6 h. The CCSD(T) calculation using 6-311 + G(d,p) basis set plus a ‘ghost atom’ H-Bq obtains Hp–*π* energy −12.887 kJ/mol, better than that of the CCSD(T)/cc-pVTZ method. However, the cpu-time of CCSD calculation is much shorter than the CCSD(T) calculation. Another advantage of using the ghost atom is reducing the basis set superposition error (BSSE) (Asturiol et al., 2008; Balabin, 2008; Mayer & Valiron, 1998).

In this study, the molecular interaction energy between two molecules A and B are defined as the energy difference between A andB complex and the molecular monomers A and B; i.e.
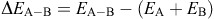



Negative interaction energy values represent the attractive interactions, and the positive interaction energy values indicate the repulsive interactions. In this study, the large-scale scanning calculations are performed using CCSD/6-311 + G(d,p) method, and the interaction energies at optimized structures are calculated using the most advanced method CCSD(T)/6-311 + G(d,p)+H-Bq. The ‘ghost hydrogen atom’ (H–Bq) is attached to the polar hydrogen atom, and the distance to polar hydrogen atom is 0.8 Å. Keep it in mind that in the calculations for Hp–*π* interaction energies, the same ghost atom is also added to the two molecule monomers. The Hp–*π* interaction energies in solutions are calculated using the polarizable continuum model (PCM) (Amovilli et al., 1999; Cossi & Barone, 1998; Foresman, Keith, Wiberg, Snoonian, & Frisch, 1996; Miertuš, Scrocco, & Tomasi, 1981). In PCM method, the solvation effect of protein in solution is modeled as a polarizable continuum, rather than individual molecules, makes *ab initio* computation feasible. All calculations were carried out with the Sugon-5000A computer and TH-1A super computer (www.nscc-tj.gov.cn) using Gaussian 09 and Gauss View 5 software packages (Frisch et al., 2010).

## 3. Results

All the results obtained are reported and summarized using tables and figures, followed up by brief comparisons and illustrations.

### 3.1. Hp–π interactions in protein backbones

**Figure 3.**
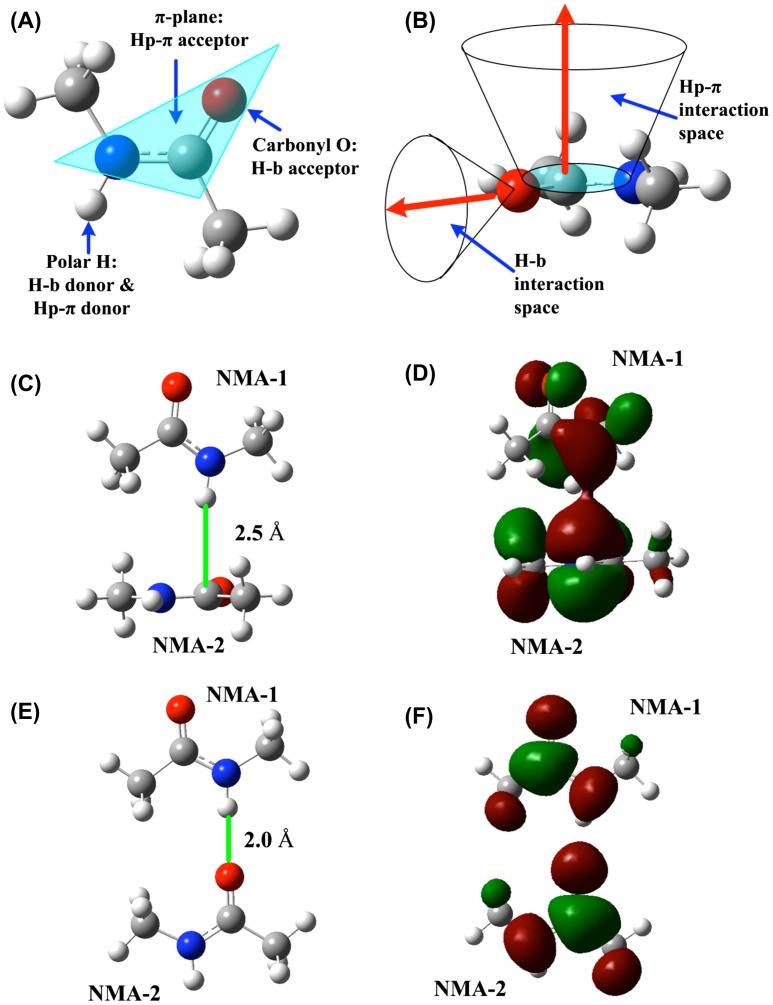
The structure of NMA is the model of peptide bond unit in protein backbones. (A) In NMA, the atom N, C, and O comprise a *π*-plane, possessing the interaction elements for both common hydrogen bond interactions and for polar hydrogen–*π* interactions. The polar hydrogen atom, attaching on the nitrogen atom, is the donor for both common hydrogen bonds and polar hydrogen–*π* bonds. The carbonyl oxygen atom is the acceptor of common hydrogen bonds, and the N–C–O triangle *π*-plane is the acceptor of the polar hydrogen–*π* interactions. (B) The interaction spaces of common hydrogen bonds and of the Hp–*π* interactions in protein backbones. The common hydrogen bond interaction space is in the front of oxygen atom on the *π*-plane, and the Hp–*π* interaction space is on the top of the N–C–O triangle *π*-plane. (C) The polar hydrogen–*π* interaction (Hp–*π*) structure between two NMA molecules. The polar hydrogen atom of NMA-1 perpendicularly points to the *π*-planes of the NMA-2. (D) The HOMO of Hp–*π* interaction system. The polar hydrogen atoms are in closely touching with the NMA *π*–MO. (E) The common hydrogen bond (H–b) interaction structure between two NMA molecules. (F) The HOMO of H-b interaction between two NMA molecules.

The peptide bond unit in protein backbones can be represented by the *n*-methyl acetamide (NMA), in which the atom N, C, and O compose a *π*-plane, with the elements for both common hydrogen bond interactions and the polar hydrogen–*π* interactions, as shown in Figure 3(A). The polar hydrogen atom, attached to the nitrogen atom, is the donor of both common hydrogen bonds and the polar hydrogen–*π* bonds. The carbonyl oxygen atom is the acceptor of common hydrogen bonds, and the N–C–O triangle *π*-plane is the acceptor of the polar hydrogen–*π* interactions. In the protein backbone structures, the interaction space for hydrogen bond interaction is in the front of oxygen atom on the *π*-plane, and the Hp–*π* interaction space is on the perpendicular direction of the N–C–O triangle *π*-plane, as shown in Figure 3(B). The interaction space of Hp–*π* bond is much larger than that of hydrogen bond interactions. The Hp–*π* interaction structure, between two NMA molecules is shown in Figure 3(C). In the Hp–*π* interaction structure the polar hydrogen atom of NMA-1 is perpendicularly pointing to the *π*-plane of NMA-2. The HOMO of Hp–*π* interaction is illustrated in Figure 3(D), in which the polar hydrogen atom of NMA-1 is in closely touching with the *π*-MO of peptide bond unit. The main physical contributors in the Hp–*π* interactions are the electrostatic interaction and the molecular orbital coordination between the polar hydrogen atom and the *π*-MO of conjugate molecules (Du, Long, Meng, & Huang, 2012). Shown in Figure 3(E) and (F) is a comparison between the two NMA molecules in the common hydrogen bond interaction structure.

The interaction energies of some typical Hp–*π* interactions are listed in Table 2. Comparing the Hp–*π* interaction energies of NMA–C_2_H_4_ (−12.887 kJ/mol) with NMA–C_6_H_5_CH_3_ (−22.731 kJ/mol), we can see that the Hp–*π* energies increase with the size of conjugate (or aromatic) molecules. The Hp–*π* interactions are the point to *π*-plane interactions, which could happen at all *π*-plane. In the NMA–NMA Hp–*π* interaction, the interaction energies at three atoms N, C, O, and at the mass center of *π*-plane are calculated individually. For facilitating comparison, in Table 2 we also list the interactions energies of the cation–*π* interaction CH_3_NH_3_^+^–C_6_H_5_CH_3_ (−50.86 kJ/mol) (Du, Long, et al., 2012; Du, Meng, Liao, & Huang, 2012), the hydrogen bond energies of water–water (−21.26 kJ/mol) and NMA–NMA, (−24.39 kJ/mol). In the hydrogen bond interaction of NMA–NMA the two *π*-planes share the same plane, and the polar hydrogen atom of NMA-1 points to the carbonyl oxygen atom of the NMA-2. The cation–*π*n–*π* interaction energy (−50.86 kJ/mol) of CH_3_NH_3_^+^–C_6_H_5_CH_3_ is much larger than the energies of three Hp–*π* interactions because of the large positive charge of proton than the charge of polar hydrogen atom. The NMA–NMA Hp–*π* interaction energies at N (−17.33 kJ/mol), C (−16.46 kJ/mol), and the mass center (−16.00 kJ/mol) are around 2/3 of the NMA–NMA hydrogen bond energy (−24.39 kJ/mol), and the Hp–*π* energy at O (−24.25 kJ/mol) is very close to the hydrogen bond energy of NMA–NMA. The Hp–*π* interaction energies are comparable to the common hydrogen bonds, therefore are one of the main contributors that maintains the structures of protein backbones. In Table 2 the Hp–*π* interaction energies of NMA–NMA in water solution are calculated using the PCM (Amovilli et al., 1999; Cossi & Barone, 1998; Foresman et al., 1996; Miertuš et al., 1981), which are around half of the Hp–*π* interaction energies in vacuum. Like common hydrogen bond interactions, the Hp–*π* interactions are less affected by the solvation effect and play significant roles in the aqueous solutions of proteins.

**Table 2.** The interaction energies^a^ and geometry parameters of some typical Hp–*π* interactions, cation–*π* interactions, and hydrogen bond interactions.

Interaction	Molecule pair	Energy (kJ/mol)	Distance (Å)
	NMA–C_2_H_4_	−12.887	2.535
	NMA–C_6_H_6_	−22.731	2.405
Hp–*π*	^b^NMA–NMA	in vacuum	
in vacuum	NMA–N	−17.329	2.310
	NMA–C	−16.464	2.514
	NMA–O	−24.252	2.368
	NMA-Center	−16.001	2.550
	^b^NMA–NMA	in water	
Hp–*π*	NMA–N	−12.207	2.654
in water	NMA–C	−8.276	3.110
	NMA–O	−15.759	2.319
Cation–*π*	CH_3_NH_3_^+^–C_6_H_6_	−50.860	3.140
H-bond	^c^NMA–NMA	−24.391	2.019
	H_2_O–H_2_O	−21.258	1.997

^a^ Hp–*π* interaction energies are calculated at CCSD(T)/6-311 + G(d,p)+H-Bq level.

^b^ In the Hp–*π* interaction the polar hydrogen atom of NMA-1 perpendicularly points to the N, C, and O of NMA-2, respectively.

^c^ In the common hydrogen bond between two NMAs, the two *π*-planes are in parallel.

### 3.2. Potential energy surface of NMA–NMA Hp–π interaction

**Figure 4.**
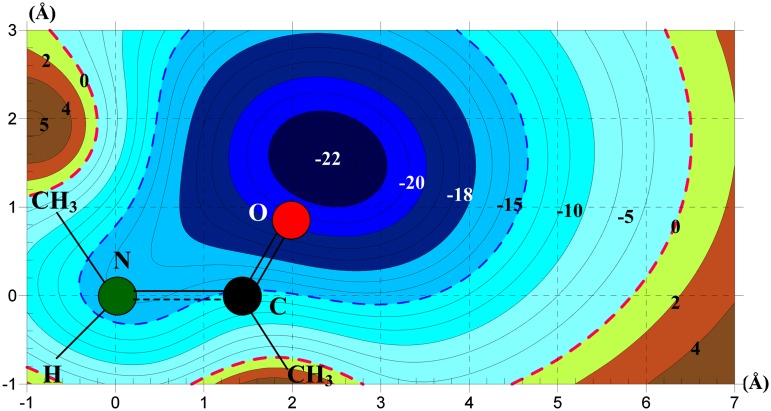
The potential energy surface of NMA–NMA Hp–*π* interactions. The NMA–NMA Hp–*π* interaction potential surface is like sliding board, where a potential energy groove is in the N–C–O triangle *π*-plane. The potential energies at three atoms N, C, and O are −17.329, 16.464, and −24.252 kJ/mol, respectively. In the calculations the polar hydrogen atom of NMA-1 keeps perpendicular direction to the *π*-plane of NMA-2.

As illustrated in above section, the Hp–*π* interactions are the point to *π*-plane interactions, which could occur at all *π*-plane. It is helpful to scan the potential energy surface of NMA–NMA Hp–*π* interactions at the *π*-plane. The scanning calculations are performed in a 4 × 8 Å^2^ area at the *π*-plane with a step of 0.1 Å increment. A total of 3321 points were computed, which was a computation intensive and cpu-time consuming procedure. In the scanning calculations, the distance between the polar hydrogen atom and the *π*-plane was kept at 2.5 Å that was the typical Hp–*π* interaction distance. The contour map of potential energy surface of NMA–NMA Hp–*π* interactions is shown in Figure 4.

In general, the potential energy surface of NMA–NMA Hp–*π* interaction is like a curve sliding board, where a potential energy groove is along the chemical bond direction N→C→O, and bounded by potential energy barriers. In the potential energy surface, there are three narrow potential energy wells at the positions of N (−17 kJ/mol), C (−16 kJ/mol), and O (−24 kJ/mol), and a broad potential energy pool at the front of O atom. The Hp–*π* interaction energies of NMA–NMA are listed in Table 2. In the process of calculation, the polar hydrogen was kept perpendicular direction to the *π*-plane. If the hydrogen atom changed its direction gradually, and eventually went to the *π*-plane of NMA-2, the NMA–NMA Hp–*π* interaction would change to NMA–NMA hydrogen bond interaction. In this case, the fourth broad energy well might spread even deeper and broader.

### 3.3. Potential energy surface scanning for torsion angles Φ and Ψ

**Figure 5.**
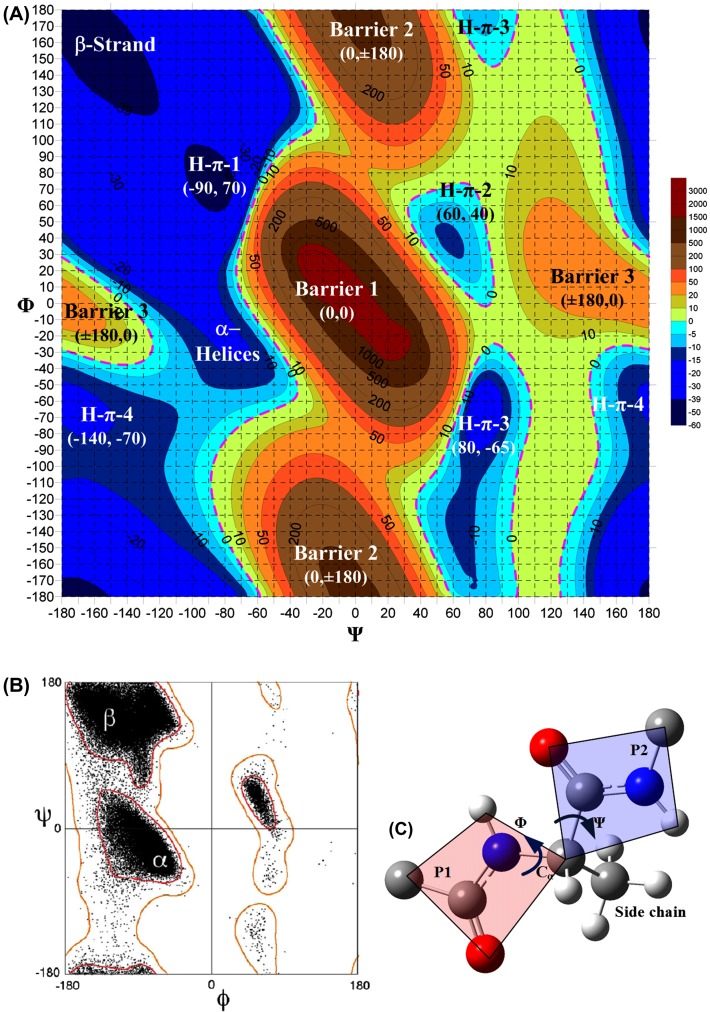
Four loop structural conformations and corresponding Hp–*π* interaction types (H–*π*–1, H–*π*–2, H–*π*–3, and H–*π*–4) in dipeptide Φ–Ψ potential map. (A) Four Hp–*π* conformations distribute in the four quadrants of the potential map. The H–*π*–1 conformation is in the up-left quadrant, centered in the position (Φ = −90°, Ψ = 70°). The H–*π*–2 conformation is in the up-right quadrant, centered in the position (Φ = 60° and Ψ = 40°), bounded by the −10 kJ/mol contour line. The center of H–*π*–3 conformation is in the lower-right quadrant, located in the (Φ = 80° and Ψ = −65°) area. The fourth Hp–*π* conformation (H–*π*–4) is in the lower-left quadrant, centered in the area (Φ = −140° and Ψ = −70°). (B) The Ramachandran plot (http://en.wikipedia.org/wiki/Ramachandran) was generated using density-dependent smoothing for 81,234 non-Gly, non-Pro, and non prePro residues with *B* < 30 from 500 high-resolution proteins (45). The Φ–Ψ contour map of theoretical potential energy surface is basically consistent with the Φ-Ψ torsion conformations in Ramachandran plots. (C) The definition of two torsion angles Φ and Ψ. The two connected peptide bond units are denoted by P1 and P2, respectively.

Shown in Figure 4 is the potential energy scanning of NMA–NMA Hp–*π* interactions, where the two NMAs are independent molecules, which cannot be directly used to illustrate the backbones of proteins, because the peptide bond units in protein backbones are connected by the single bonds of the C_α_ atoms. Between the two connected peptide bond units (denoted by p1 and p2), there are many possible combinations of the two torsion angles Φ (rotation along the N–C_α_ bond) and Ψ (rotation along the C_α_–C bond) (Lovell et al., 2003; Perczel, Farkas, Jákli, Topol, & Csizmadia, 2003; Wi & Spano, 2011). The connected peptide bond units and the definition of torsion angles Φ and Ψ are shown in Figure 5(C).

A potential energy surface scanning for the torsion angles Φ and Ψ is very helpful for exploring the full aspects of the Hp–*π* interactions in protein backbones. Shown in Figure 5(A) is the Φ–Ψ contour map of potential energy surface between two connected peptide bond units from −180° to 180° for both Φ and Ψ in 5° increment. The potential energy barriers, wells, and platforms in the Φ–Ψ contour map are summarized and illustrated in Table 3. The Φ–Ψ contour map is separated by three high-potential energy barriers from north to south in the middle of the map. The highest mountain peak in the center of the map is the conformation (Φ = 0°, Ψ = 0°), in which the carbonyl oxygen atom of p1 is in conflict with the polar hydrogen atom of p2, as shown in Table 3. The other two energy barriers (at the top and bottom), actually, are the same conformation (Φ = 0°, Ψ = ±180°), in which the two carbonyl oxygen atoms of p1 and p2 are very close. The two smaller energy barriers in the middle left and right are in the same conformation (Φ = ±180°, Ψ = 0°), in which the two polar hydrogen atoms of p1 and p2 are very close to each other (see Table 3).

**Table 3.** The structures and energies of some key interaction conformations in potential energy contour map of Φ-Ψ torsion angle scanning.

^a^Energy barrier 1		Energy barrier 2	
	Φ = 0°, Ψ = 0°		Φ = 0°, Ψ = ±180°
	Δ*E* = ∞		Δ*E* = 814.6521
	Collision between H and O		Repulsion between two O atoms
Energy barrier 3		*β*-Sheet	
	Φ = ±180°, Ψ = 0°		Φ = −165°, Ψ = 165°
			2.14 Å
	Δ*E* = 38.2561		Δ*E* = −46.0318
	Repulsion between two H atoms		Most stable inner H-b
^b^*α*-Helices		H–*π*–1	
	Φ = −65°, Ψ = −40°		Φ = −90°, Ψ = 70°
			R_H–O_ = 2.38 Å
	Δ*E* = −16.0318		Δ*E* = −43.4913
	Overlapped by H–*π*–1		H–*π* bond
			Loop
H–*π*–2		H–*π*–3	
	Φ = 60°, Ψ = 40°		Φ = 80°, Ψ = −65°
	R_H–N_ = 2.31 Å		R_H–O_ = 2.03 Å
	Δ*E* = −12.9928		Δ*E* = −23.9234
	H–*π* bond		H–*π* bond
	Loop		Loop
H–*π*–4		^c^Reference state	
	Φ = −140°, Ψ = −70°		Φ = 90°, Ψ = 180°
	R_H–C_ = 2.51 Å		
	Δ*E* = −14.6219		Δ*E* = 0.0000
	H–*π* bond		Unfavorable for H–*π* interactions
	Loop		

^a^ Energies in kJ/mol.

^b^ The structural conformations of *α*-Helices and Hp–*π* bonds (H–*π*–1) partially overlap each other.

^c^ The interaction energies (Δ*E*) of different interaction conformations are the energy differences between the energies of interaction conformations and the energy of a reference state. In this study, an unfavorable configuration (Φ = 90° and Ψ = 180°) for the Hp–*π* interactions is selected as the reference state.

The Φ-Ψ conformations of *β*-strand are concentrated in the up-left corner (Φ = −180° and Ψ = 180°, deep blue). Actually, the other three corners are the same conformation (Φ = ±180° and Ψ = ±180°). In the area from (Φ = −180° and Ψ = 180°) to (Φ = −135° and Ψ = 135°) is the conformations for *β*-strands. The most stable *β*-strand conformation is (Φ = −165° and Ψ = 165°, Δ*E* = −46.03 kJ/mol).

The conformation of *α*-helices is in the region from (Φ = −90°, Ψ = −15°) to (Φ = −35°, Ψ = −70°), centered at (Φ = −65°, Ψ = −40°), keeping the appropriate 105° curve for the shape of helices. Actually, the conformations of *α*-helices are overlapped by the Hp–*π* conformations of loops. We will discuss this problem in more detail below.

In the Φ–Ψ contour map, there are four Hp–*π* conformations, located in the four quadrants of the map. In Figure 5 and Table 3 the four Hp–*π* conformations are denoted by H–*π*–1, H–*π*–2, H–*π*–3, and H–*π*–4, respectively. The H–*π*–1 conformation is in the up-left quadrant, centered in the position (Φ = −90°, Ψ = 70°, deep blue), in which the polar hydrogen atom of p2 is perpendicular to the *π*-plane of p1, and the distance between polar hydrogen atom and oxygen atom is 2.38 Å, a stable Hp–*π* bond forming (Δ*E* = −43.49 kJ/mol). The H–*π*–1 is one of the most stable conformations in the potential energy map, extending to a broad area shown in blue color (−20 kJ/mol) in Figure 5, covering the conformation of *α*-helices (Φ = −65° and Ψ = −40°) and *β*-strands (Φ = −165° and Ψ = 165°).

The H–*π*–2 configuration is in the up-right quadrant, centered in the position (Φ = 60° and Ψ = 40°), bounded by the −10 kJ/mol contour line. In the H–*π*–2 conformation, the polar hydrogen atom of p2 perpendicularly points to the nitrogen atom of p1, forming a Hp–*π* bond (Δ*E* = −12.993 kJ/mol, R_H–N_ = 2.31 Å). The center of H–*π*–3 configuration is in the lower-right quadrant, located in the (Φ = 80° and Ψ = −65°) area. In the H–*π*–3 configuration, the polar hydrogen atom of p2 perpendicularly points to the oxygen atom of p1, forming a stable Hp–*π* bond (Δ*E* = −23.923 kJ/mol, R_H–O_ = 2.03 Å).

The fourth Hp–*π* configuration (H–*π*–4) is in the lower-left quadrant, centered in the area (Φ = −140° and Ψ = −70°). Unlike other three Hp–*π* configurations, in the H–*π*–4 conformation, the Hp–*π* bond is formed between the polar hydrogen atom of p1 and the carbonyl carbon atom of p2. In the H–*π*–4 configuration, the Hp–*π* bond is in a four-atom ring. In Table 3, the curve shapes of the four Hp–*π* configurations are indicated by green dashed lines.

In the Φ–Ψ scanning calculations, a methyl (–CH_3_) is attached to the C_α_, which is the side chain of alanine. If other side chains are used, we may get different Φ–Ψ contour maps. The interaction energies of different Hp–*π* conformations are the energy differences between the energies of Hp–*π* conformations and the energy of a reference state. In this study, we select an unfavorable configuration (Φ = 90° and Ψ = 180°) to the Hp–*π* interactions as the reference state, in which the polar hydrogen atom of p2 is in the opposite direction of the p1 *π*-plane. The structure of reference state is listed in the last cell of the Table 3.

### 3.4. Comparison between Φ–Ψ potential map and Ramachandran plot

The Ramachandran plot (Ramachandran, Ramakrishnan, & Sasisekharan, 1963) was first developed in 1963 by Ramachandran et al. that shows the backbone dihedral angles Ψ against Φ of amino acid residues in protein structure. The original Ramachandran plot was generated using hard-sphere calculations. Lately, such Φ–Ψ maps of dipeptide conformations were drawn based on the high-resolution protein structures (Lovell et al., 2003; Ting et al., 2010). The Ramachandran plot (http://en.wikipedia.org/wiki/Ramachandran) in Figure 5(B) was generated using density-dependent smoothing for 81,234 non-Gly, non-Pro, and non prePro residues with *B* < 30 from 500 high-resolution proteins (Lovell et al., 2003). The Φ–Ψ contour map of theoretical potential energy surface (Figure 5(A)) is basically consistent with the Φ–Ψ torsion conformations in the Ramachandran plot (Figure 5(B)). In the Ramachandran plot, the population density of *α*-helix and *β*-strand conformations are indicated; however, there are no population distributions of loops.

The population of loops in Ramachandran plot should be larger than the population of *α*-helices and *β*-strands, because 47% residues are in the loops. Actually, the population of loops spread in broad area where the Hp–*π* conformations (H–*π*–1, H–*π*–2, H–*π*–3, and H–*π*–4) are located, and overlap part population of *α*-helices and *β*-strands. In the Ramachandran plot, the H–*π*–1 loops cover partial area of the *β*-strands in the up-left quadrant, and also overlap the area of *α*-helices between the low-left quadrant and up-left quadrant. The conformations of H–*π*–2 loops occupy the area in the up-right quadrant, and the conformations of H–*π*–3 loops are located at the area in the low-right quadrant, where is the population of the L-*α*-helices (Lovell et al., 2003). The conformations of H–*π*–4 loops spread in large area at the low-left quadrant of the Ramachandran plot, where the sparse population corresponds with the small interaction energy of H–*π*–4 interactions.

The Φ–Ψ population density in the Ramachandran plot represents the total folding free energy. However, the Φ–Ψ population density in the current theoretical Φ–Ψ potential map only represents the folding energy without taking into account the energies from other effects, such as entropy and solvation effects. A remarkable difference between the Ramachandran plot and theoretical Φ–Ψ potential map is for the *α*-helices. In the location of *α*-helices in the theoretical Φ–Ψ potential map, the potential well of *α*-helices is shallower than that of the *β*-strands. A possible reason is that more contribution from the solvent effect might be involved in forming *α*-helices than *β*-strands.

### 3.5. The Hp–π interactions in protein loops

**Figure 6.**
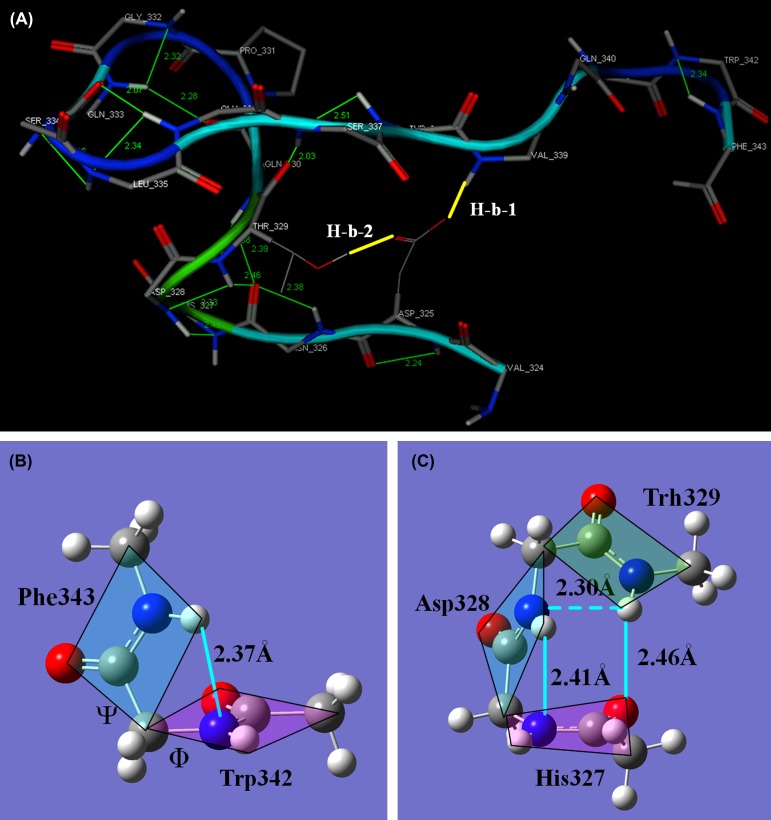
The structure of a loop (Asp325–Phe343) in *α*-amylase (pdb code: 1BLI). (A) The Hp–*π* bonds in the loop (Asp325–Phe343) of *α*-amylase 1BLI. Among the 19 residues, there are 15 Hp–*π* bonds, indicated by thin green lines. The Gln330 and Gly332 are fixed by the special peptide bonds of Pro331. The residues Thr338 and Val339 are located at the straight segment. No Hp–*π* bond supports these two residues. The Val339 and Thr329 are supported hydrogen bonds between side chain of Asp325 and peptide backbone, indicated by thick yellow lines. All other 14 residues are fixed by at least one Hp–*π* bond. (B) The Hp–*π* bond between two connected residues Trp342 and Phe343 (Φ = −60° and Ψ = −31°). (C) The Hp–*π* bonds among three continuous residues (His327, Asp328, and Thr329). Except the two Hp–*π* bonds between peptide bond unit 1 and unit 2, and between unit 2 and unit 3, the peptide bond unit 3 takes a very good orientation forming a stable Hp–*π* bond with the peptide bond unit 1.

In this section, the roles of Hp–*π* bonds in a real protein loop are analyzed. The loop in Figure 6(A) is taken from the structure of *α*-amylase (PDB code 1BLI) (Wang, Du, et al., 2009), containing 19 residues from Val324 to Phe343, in which the Asp328 is a key residue of the catalytic triad. Among the 19 residues, there are 15 Hp–*π* bonds, indicated by thin green lines. The Gln330 and Gly332 are fixed by the special peptide bonds of proline (Pro331). The residues Val339 and Gln340 are located at the straight segment of the loop, not supported by the Hp–*π* interactions. The Val339 and Thr329 are supported hydrogen bonds between side chain of Asp325 and peptide backbone, indicated by thick yellow lines. All other 14 residues are fixed by at least one Hp–*π* bond, indicated by thin green lines.

Figure 6(B) is the portrait of Hp–*π* bond between two connected residues Trp342 and Phe343. For a stable Hp–*π* bond between two connected peptide bond units, the two torsion angles Φ and Ψ have to fall in a suitable range. In Figure 6(B) the two torsion angles are Φ = −60° and Ψ = −31°. Figure 6(C) shows the Hp–*π* bonds in three continuous residues (His327, Asp328, and Thr329). The interaction geometry of the first two peptide bond units (Φ = −68° and Ψ = −16°) is the same as that in Figure 6(B), and the interaction geometry between the peptide bond unit-2 and unit-3 (Φ = −83° and Ψ = −32°) is just the same as the geometry between unit-1 and unit-2. In addition, the peptide bond unit-3 takes a very good orientation forming a stable Hp–*π* bond with the peptide bond unit-1, as shown in Figure 6(C). In this loop, a similar three-residue Hp–*π* structure is found between the continuous residues Ser334, Leu335, and Glu336.

The Hp–*π* bonds are concentrated in the curve parts of the loop. Usually, the Hp–*π* bonds act in short and continuously connected peptide bond units no more than 4 residues. In long loops, especially in the straight segment, the interactions between side chains and peptide backbones provide the supporting forces. In Figure 6(A) the side chain of Asp325 forms a stable hydrogen bond with the nitrogen atom in peptide backbone of Val 339, which is in the straight segment of the loop. Also a hydrogen bond is formed between the side chains of Asp325 and Thr329, indicated by thick yellow lines.

## 4. Discussion

In a recent paper by Kumar and Das (2012), the Hp–*π* interactions were theoretically and experimentally studied using resonant two photon ionization (R2PI), IR–UV, and UV–UV double resonance spectroscopic techniques, and high-level QM calculations. According to their experiments, the N–H…*π* hydrogen bonds and slanted T-shaped structures were observed in molecular dimer. The experimental observations could be the evidence of Hp–*π* bonds in molecular interactions. The interaction energies of polar hydrogen–*π* interactions (Hp–*π*) are much stronger than that of the non polar hydrogen–*π* interactions (H–*π*) (Du et al., 2010; Guan, Zhou, & Yan, 2012; Mróz, 1995). However, the roles of Hp–*π* interactions in protein structures have not been studied sufficiently yet.

The theoretical Φ–Ψ potential surface (Figure 5(A)) is consistent with the statistical Ramachandran Φ–Ψ plot (Lovell et al., 2003; Perczel et al., 2003; Ramachandran et al., 1963; Ting et al., 2010) to a certain extent. However, there are some subtle differences between the two plots. In Ramachandran plots, the Φ-Ψ conformation population density corresponds to the folding free energy Δ*G*, the higher population density, the lower folding free energy. On the other hand, the Φ–Ψ potential surfaces are the distribution of interaction energies Δ*E* between the two connected amino acids. The folding energies Δ*E* are the main contribution to the statistical folding free energies Δ*G*. However, there are other factors in the folding free energies, such as entropy contribution and the solvation effect. The Φ-Ψ potential surfaces cannot be exactly the same as the Ramachandran plots. The differences between two type plots may be caused by the entropy contribution and the solvation effect. The other reason is that the Ramachandran plot is the statistical results for general amino acid types (no Gly and Pro included), and the theoretical Φ–Ψ potential surface (Figure 5) is calculated from a specific amino acid alanine.

The unique properties of Hp–*π* interactions in protein loops, explored in this study may have many potential applications. A potential application of Hp–*π* interactions is in the construction of physical effective energy function (PEEF) (Balabin, 2008; Lazaridis & Karplus, 2000) to evaluate the free energies involved in protein structures and protein folding. PEEF has been used to refine X-ray crystal structures and solution NMR structures. However, these type methods generally cannot provide accurate protein folding free energies in protein design and engineering (Balabin, 2008; Lazaridis & Karplus, 2000). One reason might be the missing of Hp–*π* interactions in loops. The Hp–*π* interactions certainly can help to improve the protein structure-free energy calculations. The other potential applications of Hp–*π* interactions include the construction of protein blocks (PB) (Joseph et al., 2011; Tyagi, Bornot, Offmann, & de Brevern, 2009). Actually, the protein loops are not completely ‘irregular.’ Brevern’s group (Joseph et al., 2011; Tyagi et al., 2009) suggested that the protein structures can be classified into 16 basic construction blocks. Among them 14 PB are for loops. As shown in Figure 5(A), there are only limited Φ-Ψ conformation types (including *α*-helices, *β*-strands, and four loop types: H–*π*–1, H–*π*–2, H–*π*–3, and H–*π*–4). The basic loop types and the Hp–*π* interactions may provide a theoretical basis for deducing the folding free energies in the PB.

It is instructive to point out that, besides *α*-helix, *β*-strand and loop, the tight turn is also an important element in proteins. A tight turn in protein is defined as a site where a polypeptide chain reverses its overall direction and that the amino acid residues directly involved in forming the turn are no more than six (Chou, 2000; Richardson, 1981). According to the different manners of intra hydrogen bond interaction along a protein chain and the number of amino acid residue involved (Chou, 2000; Kabsch & Sander, 1983), tight turn can be classified into the following five types: (1) 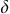 or 2-residue turn, (2) 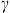 or 3-residue turn, (3) 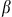 or 4-residue turn, (4) 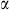 or 5-residue turn, and (5) 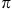 or 6-residue turn. See Equation (2) given in a comprehensive review (Chou, 2000) for a rigorous formulation in this regard. Therefore, protein loops are completely different with the tight turns as reflected by the following three facts: (1) the number of amino acid residues involved in forming a loop is much larger than that in forming a tight turn; (2) the conformation of a loop is much more irregular than that of a tight turn; and (3) the flexibility of a loop is much higher than that of a tight turn.

In the last two decades, many efforts have been made to predict tight turns, such as alpha-turn (Cai & Chou, 1999; Cai, Feng, & Li, 2003; Chou, 1997b; Pavone, Gaeta, Lombardi, Nastri, & Maglio, 1996), beta-turn, (Cai, Yu, & Chou, 1998; Chou, 1997a; Chou & Blinn, 1997; Shi, Hu, Li, & Liu, 2011; Zhang & Chou, 1997) and gamma-turn (Jahandideh, Hoseini, Jahandideh, Hoseini, & Disfani, 2009; Jahandideh, Sarvestani, Abdolmaleki, Jahandideh, & Barfeie, 2007). Recently, a unified model (Song et al., 2012) was proposed for simultaneously predicting all the five types of tight turns. However, all the aforementioned prediction methods in identifying tight turns and their types were developed by machine-learning approaches and statistical principle rather than energetics. Thus, a natural question might be raised as asking: Can the new molecular force also provide us more insights into the tight turns? This is indeed a very intriguing question. Since tight turns are completely different from loops, however, to address this problem, we need a large amount of calculations and a lot of in-depth analyses. We will investigate this in our future research.

## 5. Conclusion

Summarizing in short words, in protein backbones, when the *π*-planes of peptide bond units are in parallel orientation, they may form the common hydrogen bonds that are the supporting force in *α*-helices and *β*-strands, and when the *π*-planes are in perpendicular direction, they may form the Hp–*π* bonds that are the force maintaining the shape of loops. The important role of Hp–*π* interactions in protein folding is supported by the Ramachandran Φ–Ψ plots. The Hp–*π* interactions may provide useful theoretical concept and are helpful for development of computational methods evaluating the folding free energies of loops in protein structures. The basic PB and the structural limitations in protein folding may be derived and illustrated using the concept of Hp–*π* interactions.

Some useful concluded points can be drawn as follows. (1) Peptide bond units possess the interaction donors and acceptors of both hydrogen bond interactions and Hp–*π* bond interactions. The most unfavorable direction to the H–b interactions (perpendicular to *π*-plane) is just the most favorable direction to the Hp–*π* interactions. (2) In proteins, the energies of Hp–*π* interactions are within the range −16 to −24 kJ/mol, the bond lengths are around 2.5 Å (from polar hydrogen to *π*-plane). The Hp–*π* interaction space is larger than that of common hydrogen bond interactions. (3) The H–b interactions are the supporting force in *α*-helices and *β*-strands, and the Hp–*π* interactions are the force maintaining the structural shapes of protein loops. (4) The Hp–*π* bonds are point to *π*-plane interactions, acting on a larger area and possessing broader energy range. Consequently, the loops have more structural conformations, and more flexible structures than that of the *α*-helices and *β*-strands. (5) In water solution, the interaction energies of Hp–*π* interactions are only little smaller than in vacuum. Similar to the common hydrogen bond interactions, the Hp–*π* interactions are less affected by the solvent dielectric constants.

## Competing interests

We declare that there are no any competing interests.
